# PyPCN: protein contact networks in PyMOL

**DOI:** 10.1093/bioinformatics/btad675

**Published:** 2023-11-06

**Authors:** Serena Rosignoli, Luisa di Paola, Alessandro Paiardini

**Affiliations:** Department of Biochemical Sciences “A. Rossi Fanelli”, Sapienza University of Rome, 00185 Rome, Italy; Unit of Chemical-Physics Fundamentals in Chemical Engineering, Department of Engineering, Università Campus Bio-Medico di Roma, 00128 Rome, Italy; Department of Biochemical Sciences “A. Rossi Fanelli”, Sapienza University of Rome, 00185 Rome, Italy

## Abstract

**Motivation:**

Protein contact networks (PCNs) represent the 3D structure of a protein using network formalism. Inter-residue contacts are described as binary adjacency matrices, which are derived from the graph representation of residues (as α-carbons, β-carbons or centroids) and Euclidean distances according to defined thresholds. Functional characterization algorithms are computed on binary adjacency matrices to unveil allosteric, dynamic, and interaction mechanisms in proteins. Such strategies are usually applied in a combinatorial manner, although rarely in seamless and user-friendly implementations.

**Results:**

PyPCN is a plugin for PyMOL wrapping more than twenty PCN algorithms and metrics in an easy-to-use graphical user interface, to support PCN analysis. The plugin accepts 3D structures from the Protein Data Bank, user-provided PDBs, or precomputed adjacency matrices. The results are directly mapped to 3D protein structures and organized into interactive diagrams for their visualization. A dedicated graphical user interface combined with PyMOL visual support makes analysis more intuitive and easier, extending the applicability of PCNs.

**Availability and implementation:**

https://github.com/pcnproject/PyPCN.

## 1 Introduction

In recent years, outstanding breakthroughs have been made in the field of structural biology, thanks to the increasing amount of data obtained using innovative experimental and computational techniques (Jumper *et al.* 2021, Porta-Pardo *et al.* 2022, Burley *et al.* 2023). Although a detailed understanding of protein function and dynamics can be gained with these data, many of the unanswered questions regarding the intimate relationship between structure and function could benefit from a simplified representation of protein structural information. The graph representation of protein inter-residue contacts is an application area of graph theory termed protein contact network (PCN), which is gaining appraisal as an informative schematization of 3D protein structures (Krishnan *et al.* 2008, Di Paola *et al.* 2013). To build a PCN, the structural information of the α-carbons (Cα) is extracted to create binary matrices describing the relationships between the protein residues. The Cα are the nodes, whilst the significant contacts, according to defined thresholds, are the edges. A topological representation, in contrast to a geometric description, is considered an efficient and accurate means for structural characterization, with low computational cost (Doncheva *et al.* 2012, Tasdighian *et al.* 2014). In this regard, many of the best-known metrics for graph analyses, i.e. clustering techniques and centrality measures (Doncheva *et al.* 2011, Brysbaert and Lensink 2021), found valid application in structural biology, with proven relevance in protein allosteric, dynamic, and interaction analyses (Liu and Nussinov 2016, Das *et al.* 2021, Dubanevics and McLeish 2021, Di Paola *et al.* 2022, Guzzi *et al.* 2023).

Despite the abundance and diversity of graph analysis modules, the state of the art in the applicability of PCNs suffers from poor usability. This leads to a lack of workflows for creating and analyzing PCNs, unless there is an expert-driven integration of different tools and implementations. To address this issue and facilitate visual analysis of PCNs, some web-servers were developed in recent years (Seeber *et al.* 2015, Chakrabarty and Parekh 2016, Felline *et al.* 2020). However, despite their ease of use, the major limitation of these tools is their inherent design for specific analyses and the resulting lack of flexibility, which limits their applicability, extensibility, and integration with other protein analysis tools. On the other hand, the use of an open-source tool such as PCN-Miner (Guzzi *et al.* 2022), which offers the possibility of accessing a large number of different algorithms from a large user base, is limited by a very basic and essential interface that is not extensible and embeddable in complex analysis workflows. With this in mind, we were motivated to develop a new and more efficient environment to support PCN analysis, keeping pace with the latest modern, unified user interfaces.

**Figure 1.**
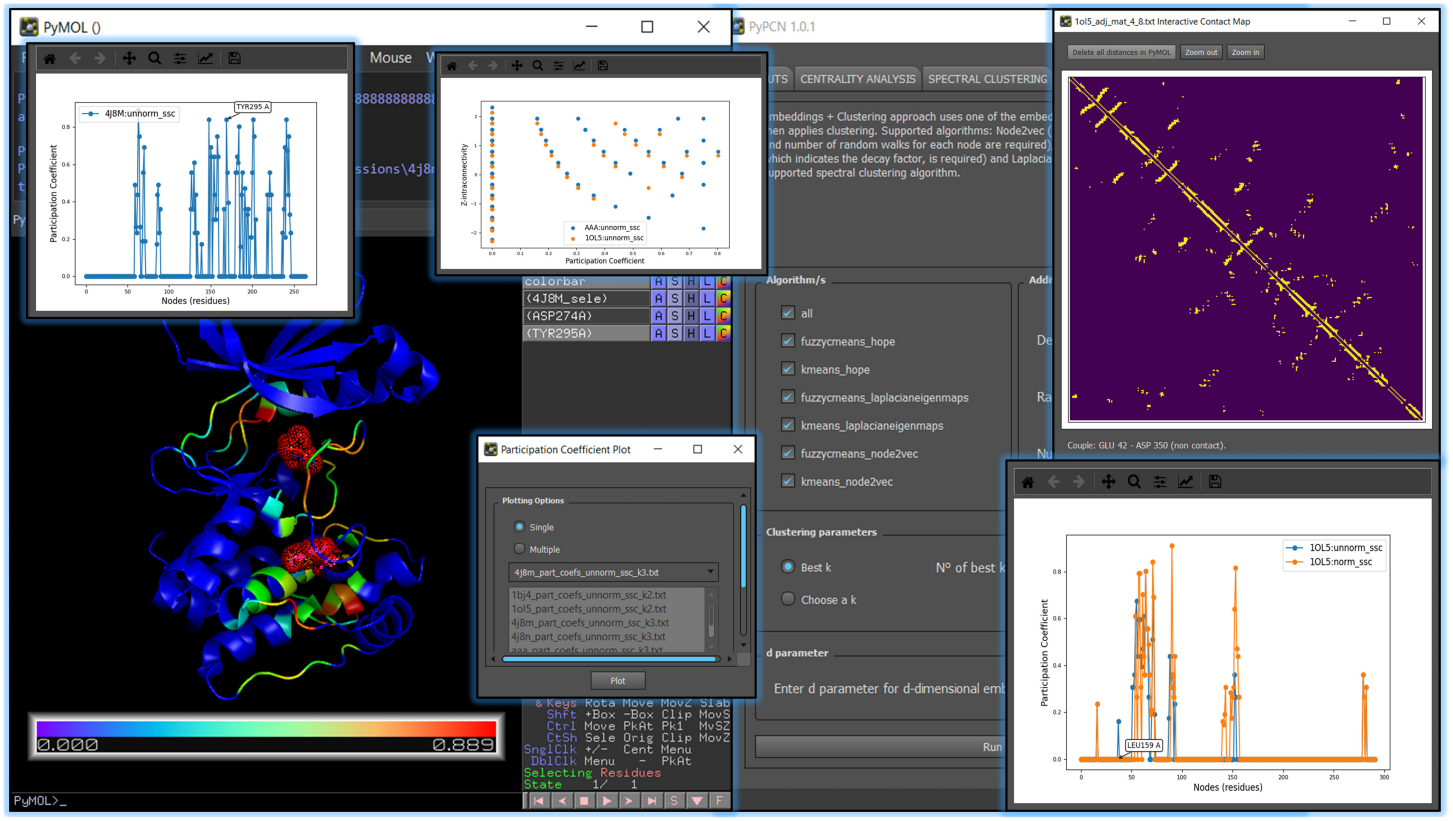
Overview of PyPCN. Some of the analyses supported by PyPCN, and its integration with PyMOL, are shown. It showcases interactive participation coefficient plots for both single structures (upper left) and multiple structures (upper right and bottom right). The upper-right window displays the interactive contact map. The PyMOL viewer demonstrates how the results are mapped onto the 3D structure, including the highlighting of residues when selected from the interactive plots. For more comprehensive information regarding all the analyses that have been implemented, refer to the User's Guide (Supplementary Material).

Here, we introduce PyPCN (Fig. 1), a user-friendly PyMOL (Schrödinger, LLC) plugin for computation, visualization and analysis of PCNs. The plugin aims to bridge the gap between knowledge of graphs and structure–function characterization of proteins, bypassing tedious command line implementations or inaccessibility of source codes for specific protocols.

## 2 Implementation

PyPCN is written in Python3 with Object-Oriented logic and the graphical user interface (GUI) development is supported by PyQT-5 (Summerfield 2008). It has been tested and works on Linux, Windows, and macOS. PyPCN works as a plugin of the popular Molecular Graphics Viewer PyMOL, which is required in its version 2.3 or higher, either Incentive or Open-source. At the first usage, the user is asked to choose a working directory, and guided to proceed with the installation of external dependencies (Supplementary Material), for which PyPCN offers an automatic process.

PyPCN computes, starting from a PDB file, the binary adjacency matrix, unless it is provided with a precomputed one in a properly formatted file (Supplementary Material). The PDB file can be provided as a valid PDB-ID in the GUI input form; a use-case in which the protein to be analyzed is downloaded from Protein Data Bank (Berman *et al.* 2000). The alternative option is to provide PyPCN for a user-customized protein structure by loading it in PyMOL.

Once the setup is complete, the workflow of the analysis is divided into 4 modules for the (i) centrality analysis; (ii) spectral clustering; (iii) embedded clustering; and (iv) community extraction. The centrality of a node (i) in PyPCN can be computed either by means of its degree of connectivity, or with path-related metrics, such as the betweenness, closeness, and eigenvector (Brinda and Vishveshwara 2005, De Ruvo *et al.* 2012, Di Paola *et al.* 2013) (Supplementary Material). With regards to the clustering and community extraction methods (ii–iv) PyPCN supports 20 algorithms and the related parameters. Along with every clustering partition, PyPCN quantifies the role of connectivity of each node (Supplementary Material), both overall and in a specific cluster, by computing the Participation Coefficient (*P*) and the Intramodular Connectivity *z*-score (*z*) (Di Paola and Giuliani 2015, Hu *et al.* 2017). Using PyPCN as an integrated platform for these algorithms serves as a foundational tool for analyzing outputs beyond the plugin's scope, i.e. graph representations of conformational ensembles (Yao *et al.* 2018).

In addition to saving the results in the previously setup working directory, PyPCN stores all information of any analysis in a dedicated window, allowing the user to conveniently proceed with multiple and comparative analysis avoiding any loss of the results.

Thanks to the implementation in PyMOL, the gap between data retrieval and visualization is filled. The per-atom computed values are color-mapped onto the 3D protein structure and straightly visualized in PyMOL. The adjacency matrices can be visualized as interactive contact maps, by clicking on which the information stored in the 2D plot is highlighted in the 3D structure. The per-residue centrality calculations, as well as *z*–*p* correlations, of one or more proteins, can be visualized as editable and interactive line plots.

## Supplementary Material

btad675_Supplementary_Data
