# Supplementary material for: PyPCN: protein contact networks in PyMOL
Source: Bioinformatics. 2023 Nov 6;39(11):btad675. doi: 10.1093/bioinformatics/btad675 (PMC10641099; doi:10.1093/bioinformatics/btad675)
Supplement: btad675_Supplementary_Data [file btad675_supplementary_data.docx]

PyPCN: Protein Contact Networks in PyMOL

User’s Guide, Quick Start, Tutorials and Supplementary Material

**GitHub repository**: **https://github.com/pcnproject/PyPCN**

**Copyright**: [2023-ongoing]:

**Last updated**: September, 2023

**Contacts:**

[serena.rosignoli@uniroma1.it](mailto:giacomo.janson@uniroma1.it)

alessandro.paiardini@uniroma1.it

**Table of Contents**

[1. Introduction 3](#_Toc146288657)

[2. Requirements 3](#_Toc146288658)

[3. Download 4](#_Toc146288659)

[4. Installation 4](#_Toc146288660)

[5. Dependencies 4](#_Toc146288661)

[6. How-to 7](#_Toc146288662)

[6.1 - Quick Guide 7](#_Toc146288663)

[6.2 - Workflow 8](#_Toc146288664)

[7. Further information 10](#_Toc146288665)

[7.1 - General information and contacts 10](#_Toc146288666)

[7.2 - How to report a bug 11](#_Toc146288667)

[7.3 - Contribute to the PyPCN development 11](#_Toc146288668)

[8. Tutorials 11](#_Toc146288669)

[8.1 - PCN on Aurora-A Kinase (PDB-ID: 4J8N) 11](#_Toc146288670)

[8.2 - Comparison of hemoglobin oxygen-bound states 13](#_Toc146288671)

[8.3 - Centroids-based PCN on Aurora-A kinase in complex with its activator TPX-2 (PDB-ID: 1OL5) 14](#_Toc146288672)

[8.4 - Conformational ensembles analysis exploiting PCNs 15](#_Toc146288673)

[9. Supporting material 17](#_Toc146288674)

[9.1 - External files formats 17](#_Toc146288675)

[9.2 - Algorithms and Metrics 17](#_Toc146288676)

[References 20](#_Toc146288677)

# Introduction

Protein Contact Networks are a way to represent the tridimensional (3D) structure of a protein, applying the typical network formalism in the description of the structure-function relationships. Inter-residue contacts are described as binary adjacency matrices, which are derived from the graph representation of protein residues (α-carbons, β-carbons or centroids) and distances according to defined thresholds. Algorithms for functional characterization are computed on binary adjacency matrices to unveil allosteric, dynamic and interaction mechanisms in proteins. Such strategies are commonly applied in a combinatorial way, albeit rarely found in seamless and user-friendly implementations.

In this context we developed PyPCN, a wrapper to several Python modules dedicated to the analyses of PCNs. PyPCN is a plugin of PyMOL, developed to support more than 20, among algorithms and metrics, hence to provide an easy-to-use Graphical User Interface (GUI) for assisting PCNs analyses. The plugin can handle the downloading of 3D structures from Protein Data Bank, user-provided PDBs or precomputed adjacency matrices. The results are directly mapped onto 3D protein structures, as well as organized for their visualization in interactive plots. A dedicated GUI, together with the visual support provided by PyMOL, makes the analysis more intuitive and simple, in a way that broadens the applicability of the analysis of proteins as PCNs.

Some general features:

- Handling of either PDBs or pre-computed adjacency matrices as inputs.
- Mapping of the results onto 3D protein structures providing an intelligible visualization.
- Support for more than 24 algorithms for PCN analyses.
- Visualization of contact matrices as interactive plots.

... and so more, see next for further details

# Requirements

**Minimal requirement:** a recent version of PyMOL installed on your computer.

PyPCN is compatible with incentive PyMOL builds distributed by [Schrodinger](https://pymol.org/2/) (required PyMOL version >= 2.3.4) and open source builds (required PyMOL version >= 2.3.0).

PyPCN is distributed freely to the public and it has been tested and runs on Windows, macOS and Linux versions of PyMOL.

Some incompatibilities may arise with the usage of PyMOL version 2.5.x if ‘undo’ function is enabled, which in PyMOL 2.5.2 still shows some shortcomings. Therefore, when the plugin is opened, the ‘undo’ function is automatically disabled and it is strongly suggested to keep it disabled when using the plugin.)

# Download

PyPCN plugin ZIP file: **https://github.com/pcnproject/PyPCN/archive/refs/heads/main.zip**

# Installation

PyPCN is installed via the PyMOL plugin manager:

- First download the latest version of the plugin ZIP file: **https://github.com/pcnproject/PyPCN/archive/refs/heads/main.zip**
- Launch PyMOL and use the *Plugin* → *Plugin Manager* command from the main menu of PyMOL. The plugin manager window of PyMOL will open.
- Click on *Install New Plugin* and press the *Choose File…* button. Select the **PyPCN ZIP file** which you have downloaded before. You will be asked to give the path of the directory in which to install the plugin files. Just select the default option if you are unsure about what to do (the location of the plugin files does not make any difference when running the plugin).

# 5. Dependencies

Some dependencies, which are not distributed along with PyMOL, must be installed. PyPCN is equipped with an **automatic installation** process, but only available on Incentive PyMOL version 2.5. After having proceeded with the installation of the external dependencies, it is necessary to close and re-open PyMOL.

Please follow the instructions reported below for those cases in which the automatic installation is not supported (i.e. Open-Source PyMOL, Incentive PyMOL versions lower than 2.5, PyMOL setups in which the automatic installation fails for not widely known reasons).

**Before continuing:** the protocol reported next is one of the multiple ways to do that, any alternative is plausible, as long as it allows for the dependencies to be correctly loaded from PyMOL (To check it, just try to import the module of interest in the PyMOL command-line).

**Manual installation protocol:**

PyMOL in Ubuntu Linux, macOS and windows operating systems can be installed in several ways. However, we would suggest installing it in a dedicated Conda environment to ensure full compatibility with the dependencies. Please, follow the steps as reported.

** The usage of a dedicated environment is suggested, but not mandatory. If you want to proceed w/o a dedicated environment, jump to step 5.

1. **Install The “Conda package manager”.**

The “Conda package manager” has two versions, Anaconda and Miniconda, which are both functional for our purposes. If the “Conda package manager” is not installed, it can be downloaded at the following links:

- Anaconda: <https://www.anaconda.com/products/distribution>
- Miniconda: <https://docs.conda.io/en/latest/miniconda.html>

1. **Create and activate a dedicated environment**

Environments in “Conda” can help in avoiding conflicts between installed packages. A documentation about that can be found here:

<https://docs.conda.io/projects/conda/en/latest/user-guide/tasks/manage-environments.html>

To create a dedicated environment, the *conda create* command should be used:

conda create --name environment_name

Then, the environment must be activated:

conda activate environment_name

1. **Install PyMOL in the environment**

When the “Conda package manager” is installed, and the dedicated environment created and activated:

- open source PyMOL can be installed as reported at the next link: <https://anaconda.org/conda-forge/pymol-open-source>
- Incentive PyMOL can be installed as reported on the official website at the next link: https://pymol.org/2/

1. **Install PyPCN**

PyPCN’s installation is the same as explained in “Section 2”.

1. **Install the dependencies**

For each dependency, type the preferred command reported in Table 5.1

OR

use the ‘requirements.txt’ file at:

https://github.com/pcnproject/PyPCN/releases/download/utilities/requirements.txt

pip install -r requirements.txt

Eventually, in a newly created environment, *Biopython* and *matplotlib* are not installed either. This lack would block the opening of PyPCN. Both are Python modules needed by some PyMOL’s features and its plugins.

*Biopython* can be installed by making use of the *conda install* command as reported at the link: <https://anaconda.org/conda-forge/biopython>

*matplotlib* can be installed by making use of the *conda install* command as reported at the link: <https://anaconda.org/conda-forge/matplotlib>

**Table 5.1:** List of external dependencies and available installation modes.

| **Name** | **Installation with pip** | **Other** |
| --- | --- | --- |
| fcmeans | pip install fuzzy-c-means |  |
| gem | pip install git+https://github.com/palash1992/GEM.git | https://anaconda.org/hcc/gem |
| cdlib | pip install cdlib | https://anaconda.org/Yquetzal/cdlib |
| pytz | pip install pytz | https://anaconda.org/conda-forge/pytz |
| node2vec | pip install node2vec | https://anaconda.org/conda-forge/node2vec |
| leidenalg | pip install leidenalg | https://anaconda.org/conda-forge/leidenalg |
| sklearn | pip install sklearn | https://anaconda.org/anaconda/scikit-learn |
| matplotlib | pip install matplotlib | https://anaconda.org/conda-forge/matplotlib |
| infomap | pip install infomap | https://anaconda.org/conda-forge/infomap |
| wurlitzer | pip install wurlitzer | https://anaconda.org/conda-forge/wurlitzer |
| karateclub | pip install karateclub | https://anaconda.org/conda-forge/karateclub |
| ASLPAw | pip install ASLPAw |  |
| Graph | pip install graph-tools | https://anaconda.org/conda-forge/graph-tool |

# 6. How-to

In this section are described the steps to be carried out for a complete usage of PyPCN. If you are looking for a quick-start please refer to *section 6.1*; whilst more detailed workflow description is provided in *section 6.2*.

## 6.1 - Quick Guide

A quick guide to learn how to use PyPCN is graphically summarized in *Figures 6.1.1-2*.

**Note:** It has been observed that in some environments the use of parallel threads is not supported, if PyMOL unexpectedly crashes during some of the processes, please try again by unchecking the option ‘Use Threads’ **[10; Figure 6.1.2]**.


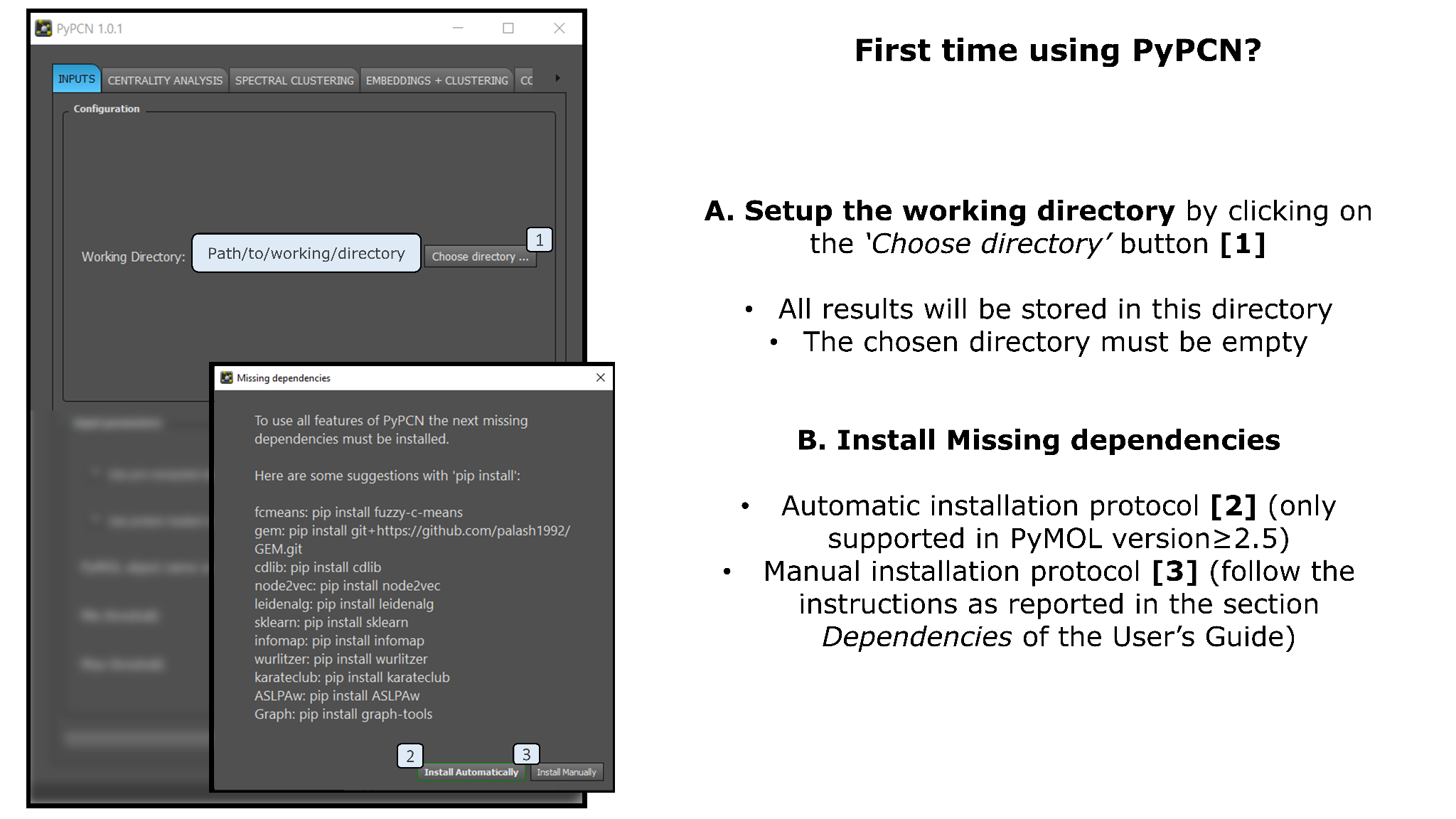


**Figure 6.1.1** Quick Guide 1 - First usage of PyPCN

**
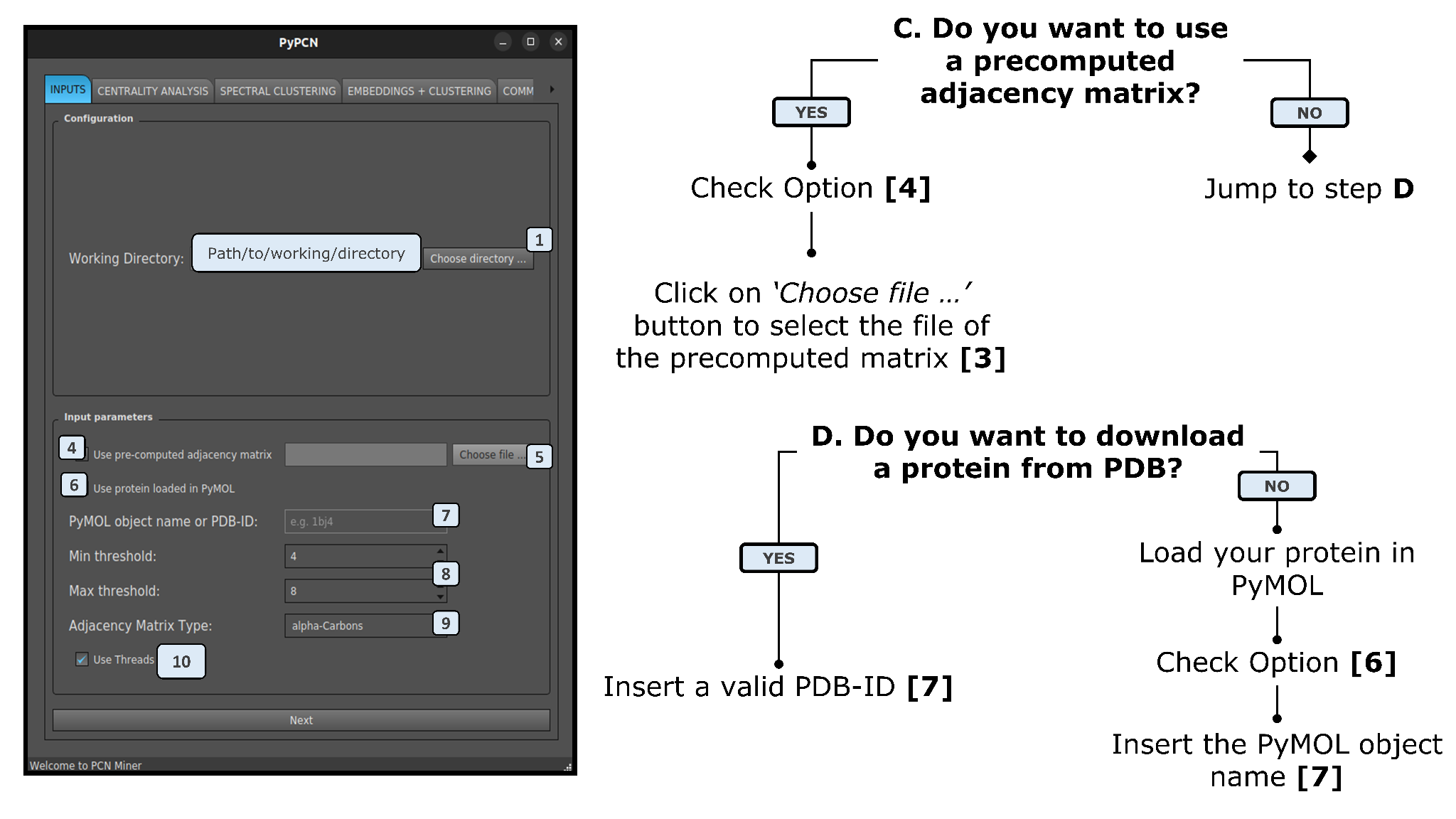
**

**Figure 6.1.2** Quick Guide - Parameters setup

## 6.2 - Workflow

1. **First time using PyPCN**

At the first usage of PyPCN, the user is asked to set up a working directory where to store all the computations. The chosen directory must be empty, otherwise, a warning message will block you from continuing further.

Once the setup is completed, the input parameters must be specified. In this case, the workflow is differentiated depending on the type of inputs.

1. **[Optional] Pre-computed adjacency matrix**

If the user has a previously computed adjacency matrix to analyze, option **[4; Figure 6.1.2]** must be checked and the *‘Choose file …’* button **[5; Figure 6.1.2]** must be used for browsing to the ‘.txt’ file in which the adjacency matrix is stored (see Section 9.1.1). Please note that in this case, PyPCN can’t be aware of the protein that is being analyzed and neither of the parameters that were used for computing the adjacency matrix. Therefore, to avoid erroneous inexplicable results: (i) the setup of Step 3 and (ii) the parameters **[8-9; Figure 6.1.2]** must correspond to the adjacency matrix in input. For related reasons, in this case the interactive contact map can’t be visualized.

1. **Protein input and adjacency matrix setup**

The protein structure to be analyzed is specified in this step. If the structure of interest is stored in the Protein Data Bank, it is sufficient to digit the PDB-ID in the line-edit **[7; Figure 6.1.2]**. This protocol supports multiple consecutive analyses by specifying different PDB-IDs in a comma-separated list, e.g. ‘1ol5, 3uoh’

Alternatively, if the input is a customized structure, option **[6; Figure 6.1.2]** must be checked and the protein must be loaded in PyMOL. In this case, the name to be specified in the line-edit **[7; Figure 6.1**.**2]** is the name of the object in PyMOL.

PyPCN supports the computation of adjacency matrices based on α-carbons, β-carbons or centroids, selected from option [**9; Figure 6.1.2**]. The threshold for occurring contacts can be selected in options [**8; Figure 6.1.2**].

1. **Algorithms selection**

The workflow of the analysis is divided into 4 modules, for which PyPCN offers a dedicated tab: (i) centrality analysis; (ii) spectral clustering; (iii) embedded clustering and (iv) community extraction. The INPUTS tab specifies common parameters for all modules, whilst the module-specific parameters can be set up in the dedicated tabs (See Section 9.2). Consecutive analyses for the same module can be run, by checking multiple options in the *‘Algorithm(s)’* section **[11; Figure 6.2.1-a]**. Click the *‘Run Analysis’* button **[12; Figure 6.2.1-a]** to proceed.

**Note:** the modules (ii-iii-iv) always compute 2 different analyses, clustering partition and participation coefficient computing, but PyMOL only loads the last one; remember to explore the Results tab (see next paragraph) to see all computations.

An additional tab, called ‘Other’, is intended to provide support for reading data from conformational ensembles statistics computed according to the Difference Contact Network Analysis (DCNA) method (<https://pubs.acs.org/doi/full/10.1021/acs.jcim.8b00250>). The full integration of DCNA features not only facilitates straightforward visualization of conformational dynamics analyses but also opens doors to the continuation of community detection and clustering analyses inherent to PyPCN. To further understand how to insert this step into your PCN analysis, please refer to the tutorial in Section 8.4. For information about the format requirements or the computations, explore, respectively, Section 9.1.2 and 9.2.

1. **Results**

The Results tab mirrors the workflow and stores all information of any computed analysis. Scroll to the analysis of interest **[13; Figure 6.2.1-b]** and use the buttons *‘Show Contact Map’* **[14; Figure 6.2.1-b; Figure 6.2.2-a]**, *‘Show Plot’* **[15; Figure 6.2.1-b]** and *‘Participation Coefficient Plot’* **[16; Figure 6.2.1-b]** to visualize the related plots, as well as the button *‘Show in PyMOL’* **[14,15]** to visualize the related results as mapped onto the 3D structure.

For the modules (ii-iii-iv) it is possible to plot the Participation Coefficient (see Section 9.2) in multiple ways **[17; Figure 6.2.1-b]**. The alternative options are, whether to plot a single or multiple analyses, and whether to plot the Participation Coefficient towards the protein residues (Figure 6.2.2-b) or the Participation Coefficient towards the intramodule-connectivity Z-score (Figure 6.2.2-c).

**Note:** all plots are interactive and by clicking on them, the information is mapped and visualized in PyMOL onto the 3D structure. When the adjacency matrix is computed according to residues’ centroids, the visualization in PyMOL is supported through the generation of pseudo-atoms at the centre of mass coordinates of the residues of interest.


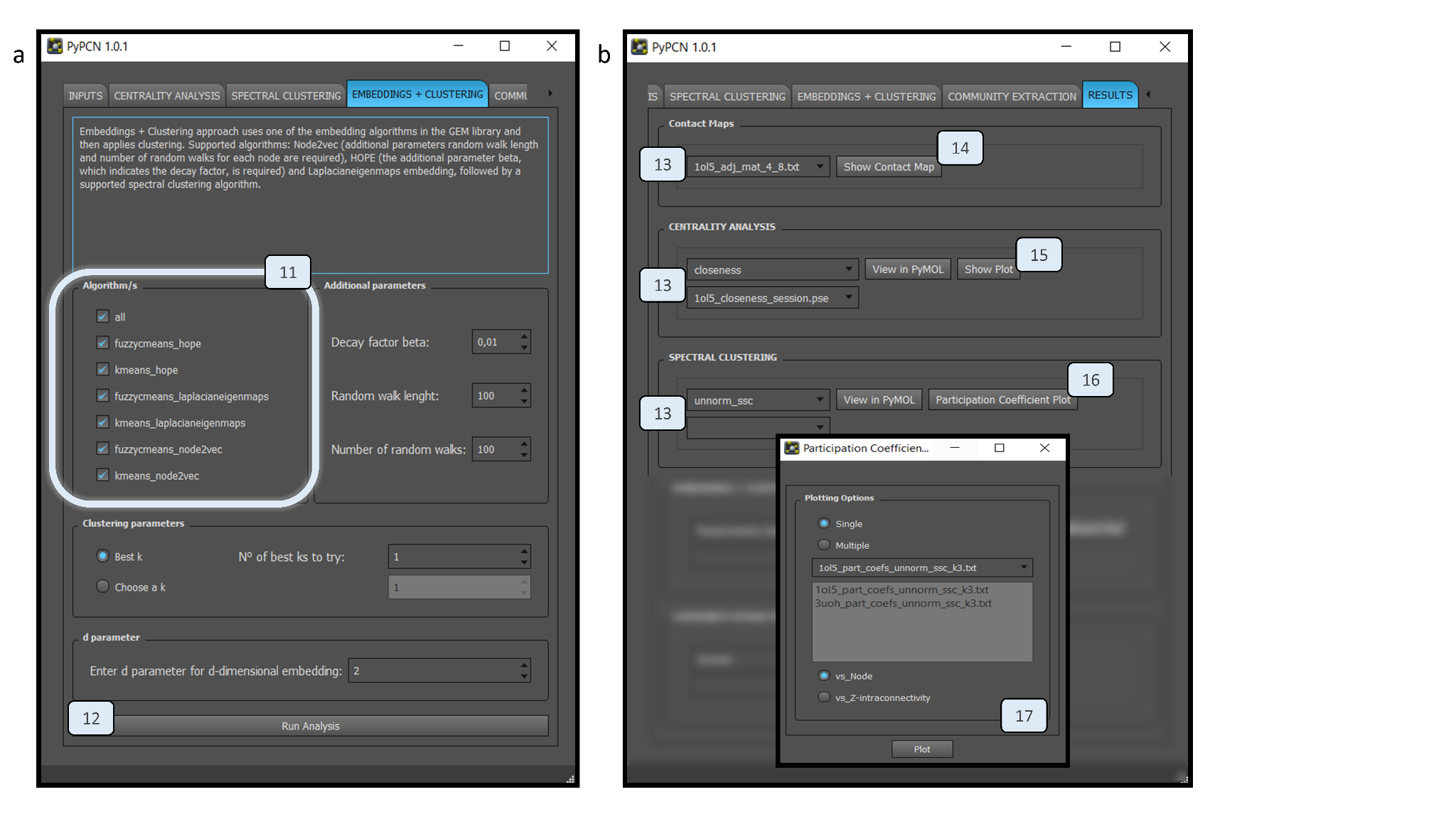


**Figure 6.2.1** Quick Guide 3 - Algorithms selection (a) and visualization of the results (b)


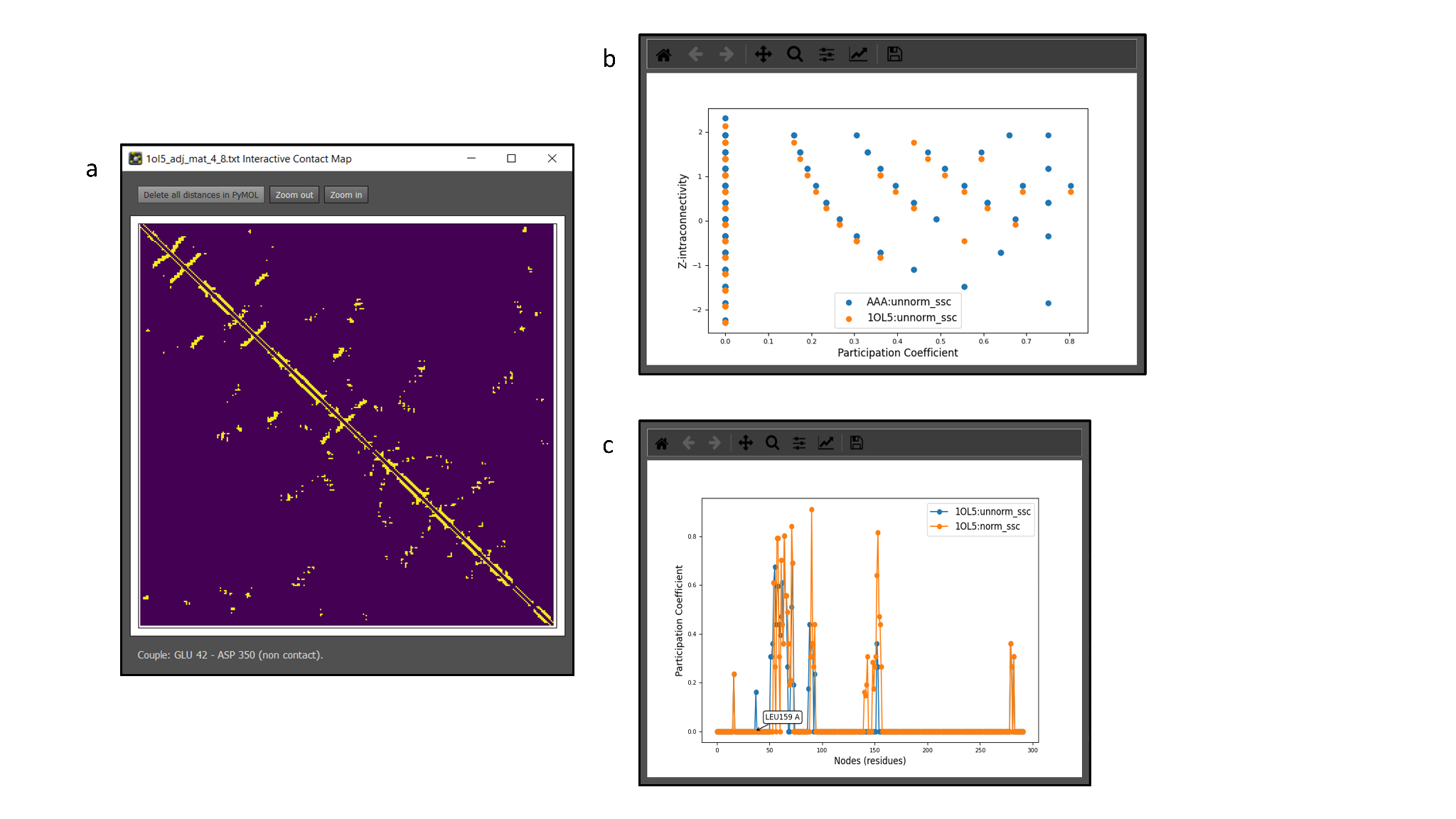


**Figure 6.2.2** Interactive plots. (a) Contact Map; (b) Participation coefficient and intraconnectivity-z-score; (c) per-residue Participation coefficient.

# 7. Further information

## 7.1 - General information and contacts

PyPCN is distributed under the **GPL-3.0 License**.

The source code is hosted on GitHub at: https://github.com/pcnproject/PyPCN

The PyPCN project is ongoing. To be up to date with bug fixes and new releases, please visit its GitHub page.

PyPCN has been tested on multiple Operating Systems and PyMOL setups to guarantee its broad applicability. In Table 7.1 is reported a list of the platforms on which we have tested PyPCN.

**Table 7.1:** List of the platforms in which we have tested PyPCN.

| **PyMOL version** | **Operating system** | **PyMOL source** |
| --- | --- | --- |
| 2.5.4 | Linux (Ubuntu 20.04.3 LTS), 64-bit | Incentive |
| 2.5.5 | Linux (Ubuntu 22.04.1 LTS), 64-bit | Incentive |
| 2.5.4 | Linux (Ubuntu 18.04.2 LTS), 64-bit | Incentive |
| 2.5.0 | Linux (Ubuntu 22.04.1 LTS), 64-bit | Open source (Conda package) |
| 2.5.0 | Linux (Ubuntu 20.04.3 LTS), 64-bit | Open source (Conda package) |
| 2.3.4 | Linux (Ubuntu 20.04.3 LTS), 64-bit | Incentive |
| 2.5.2 | Windows (v.10 Home), 64-bit | Incentive |
| 2.5.4 | Windows (v.10 Pro), 64-bit | Incentive |
| 2.5.2 | MacOS (High Sierra v.10.13.6) | Incentive |
| 2.4.0 | MacOS (High Sierra v.10.13.6) | Open source (Conda package) |
| 2.5.2 | MacOS (Monterey v.12.2.1) | Incentive |
| 2.5.2 | MacOS (Big Sur v.11.6.5) | Incentive |
| 2.4.0 | MacOS (Big Sur v.11.6.5) | Open source (Conda package) |

## 7.2 - How to report a bug

If you find a bug when using PyPCN, don’t hesitate to email us at: serena.rosignoli@uniroma1.it or alessandro.paiardini@uniroma1.it. In order to help us reproduce the problem (and to fix it more easily), a list of useful information which you should include in the email are:

- Your PyMOL version and operating system
- If you are able to run PyPCN and encounter some problem when you are using some of its functionalities, it would be really useful to us to know the input data and much more information as possible about the steps carried out.
- You may also signal the bug by opening an issue on the PyPCN GitHub repository.

## 7.3 - Contribute to the PyPCN development

If you wish to report bugs or collaborate on the development of PyPCN with suggestions for new functionalities, better documentation or improvements, you are more than welcome! Just contact us at: serena.rosignoli@uniroma1.it or alessandro.paiardini@uniroma1.it.

# 8. Tutorials

## 8.1 - PCN on Aurora-A Kinase (PDB-ID: 4J8N)

This is a use case in which we will explore the usage of PyPCN with a user-customized protein structure.

1. **Load the protein in PyMOL**

Even if we have a valid PDB-ID, we proceed by loading it in PyMOL because some modifications are needed. Type the next command in the PyMOL prompt.

> fetch 4j8n

1. **Remove additional chains**

We are interested in computing a PCN on a single monomer, hence we proceed by removing the unwanted chains.

> select "chains", chain B or chain C or chain D

> remove chains

1. **Rename PyMOL object**

To avoid confusion with the original structure of the PDB-ID 4j8n, the PyMOL object is renamed as follows:

> set_name 4j8n, "4j8n-mod"

1. **INPUTS tab (Figure 8.1.1-a)**

- Check the option *‘Use protein loaded in PyMOL’*.
- Insert the PyMOL object name (“4j8n-mod”) in the input prompt named as *‘PyMOL object name or PDB-ID’*.
- Disable ‘Use Threads’ if necessary (see Note in Section 6.1)

1. **SPECTRAL CLUSTERING tab (Figure 8.1.1-b)**

- Check one or multiple preferred algorithms. In this example we will use an un-normalized Soft Spectral Clustering method, hence the option ‘unnorm_ssc’ is checked.
- Select *‘Choose a k’* option and set the number of clusters at 2
- Click on ‘Run Analysis’

1. **RESULTS tab (Figure 8.1.1-c,d)**

After a Spectral Clustering analysis, also the Participation Coefficient is computed (as for all the modules ii-iii-iv; see Section 6.2). Therefore, two different analyses can be explored, which in this case are named as ‘4j8n-mod_part_coefs_unnorm_ssc_k2_session.pse’ and ‘ ‘4j8n-mod_Clusters_unnorm_ssc_k2_session.pse’.

Scroll to the result of interest and explore with the *‘View in PyMOL’* and *‘Participation Coefficient Plot’* buttons


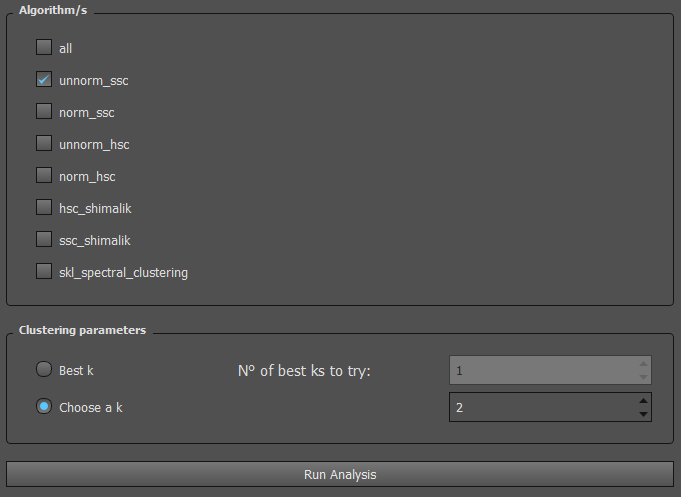


**a**

**b**

**c**


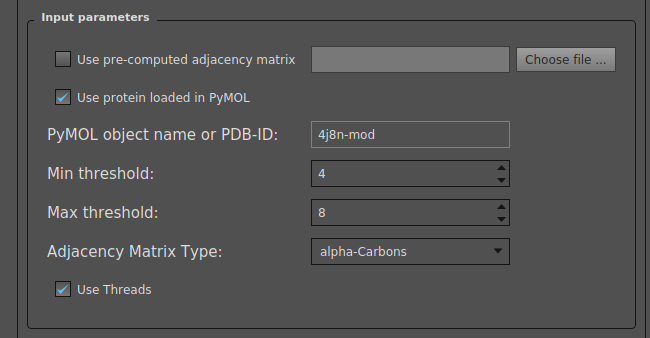

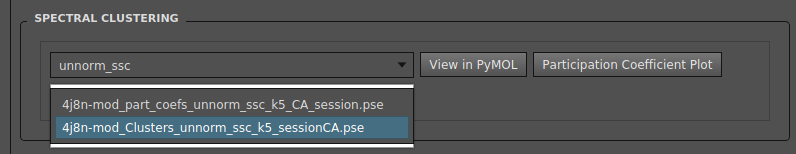

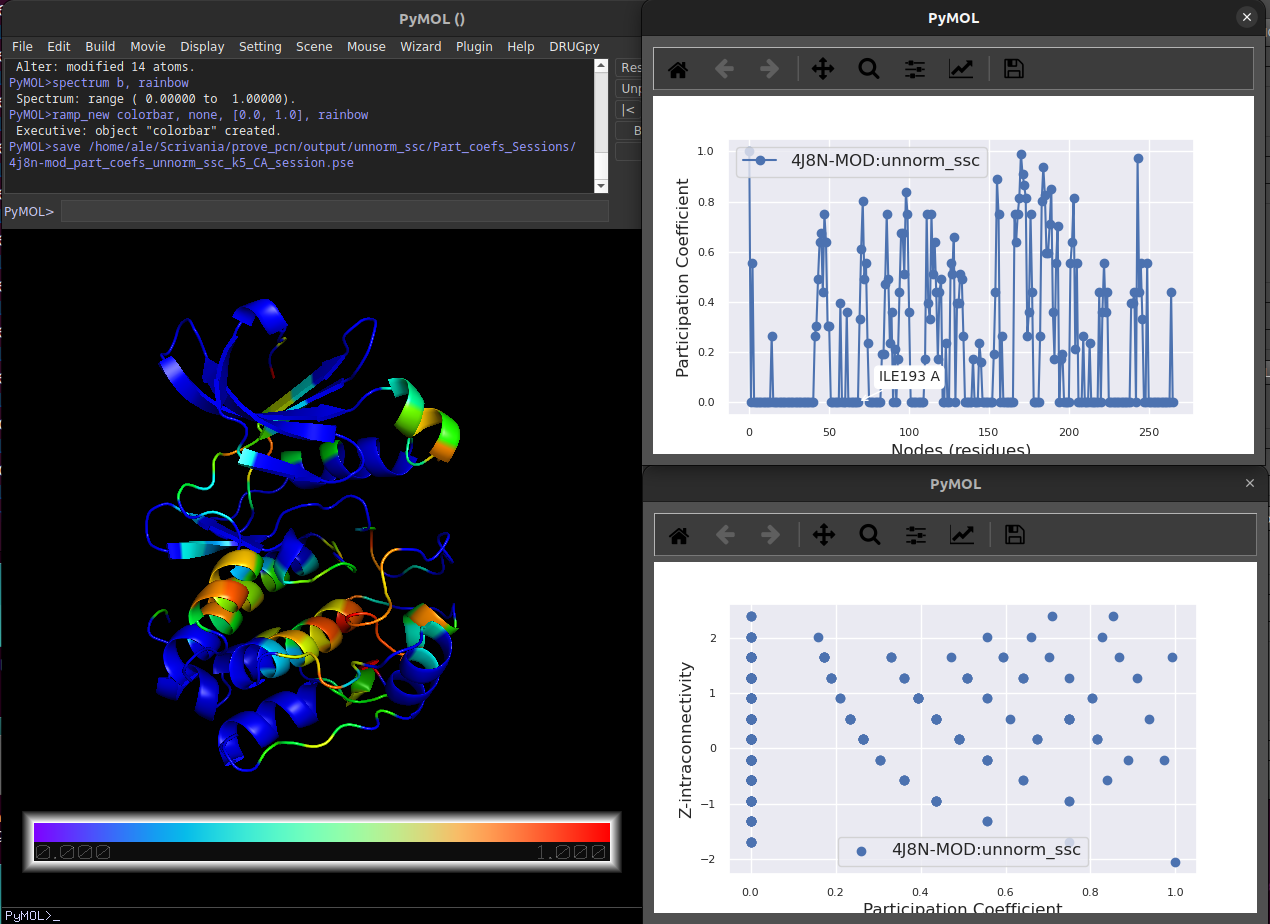


**d**

**Figure 8.1.1** Tutorial - PCN on Aurora-A Kinase (PDB-ID: 4J8N)

## 8.2 - Comparison of hemoglobin oxygen-bound states

This is a use case in which we will explore the usage of PyPCN for multiple consecutive analyses and comparisons.

1. **INPUTS tab**

The PDB-IDs of interest are: 2DN3; 2DN2; 2DN1. The PDB-IDS correspond to the protein to be analyzed, without any modification required. Therefore, we can directly Insert the PDB-IDs in a comma-separated list (“2dn3, 2dn2, 2dn1”) in the input prompt named as *‘PyMOL object name or PDB-ID’*.

1. **SPECTRAL CLUSTERING tab**

- Check one or multiple preferred algorithms. In this example we will use an un-normalized Soft Spectral Clustering method, hence the option ‘unnorm_ssc’ is checked (See Section 9.2).
- Select *‘Best k’* option and leave the other parameters in default.
- Click on ‘Run Analysis’

1. **EMBEDDINGS + CLUSTERING tab**

- Check one or multiple preferred algorithms. In this example we will use a combination of the ‘HOPE’ embedding algorithm and a ‘fuzzy-c-means clustering’ (See Section 9.2), hence the options ‘fuzzycmeans_hope’ and ‘kmeans_hope’ are checked.
- Select *‘Best k’* option and leave the other parameters in default.
- Click on ‘Run Analysis’

1. **RESULTS tab (Figure 8.2.1-b)**

After a Clustering partition, also the Participation Coefficient is computed (as for all the modules ii-iii-iv; see Section 6.2). Explore the RESULTS that are stored in the two sections ‘SPECTRAL CLUSTERING’ and ‘EMBEDDINGS + CLUSTERING’.

1. **Participation Coefficient Plot (Figure 8.2.1-c)**

To inspect how the Participation Coefficient changes upon oxygen binding, use the ‘Participation Coefficient Plot’ button that will open the window shown in Figure 8.2.1-a.

- Check ‘Multiple’
- select the analyses of the three different proteins carried out with the same algorithm
- Click on ‘Plot’
- Repeat these steps with the remaining algorithms.


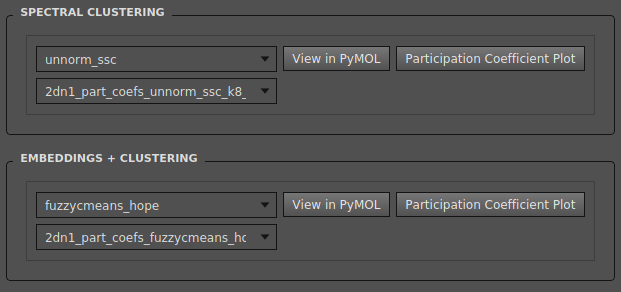


**a**

**b**


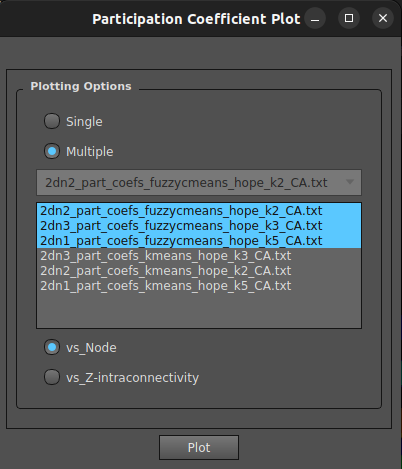

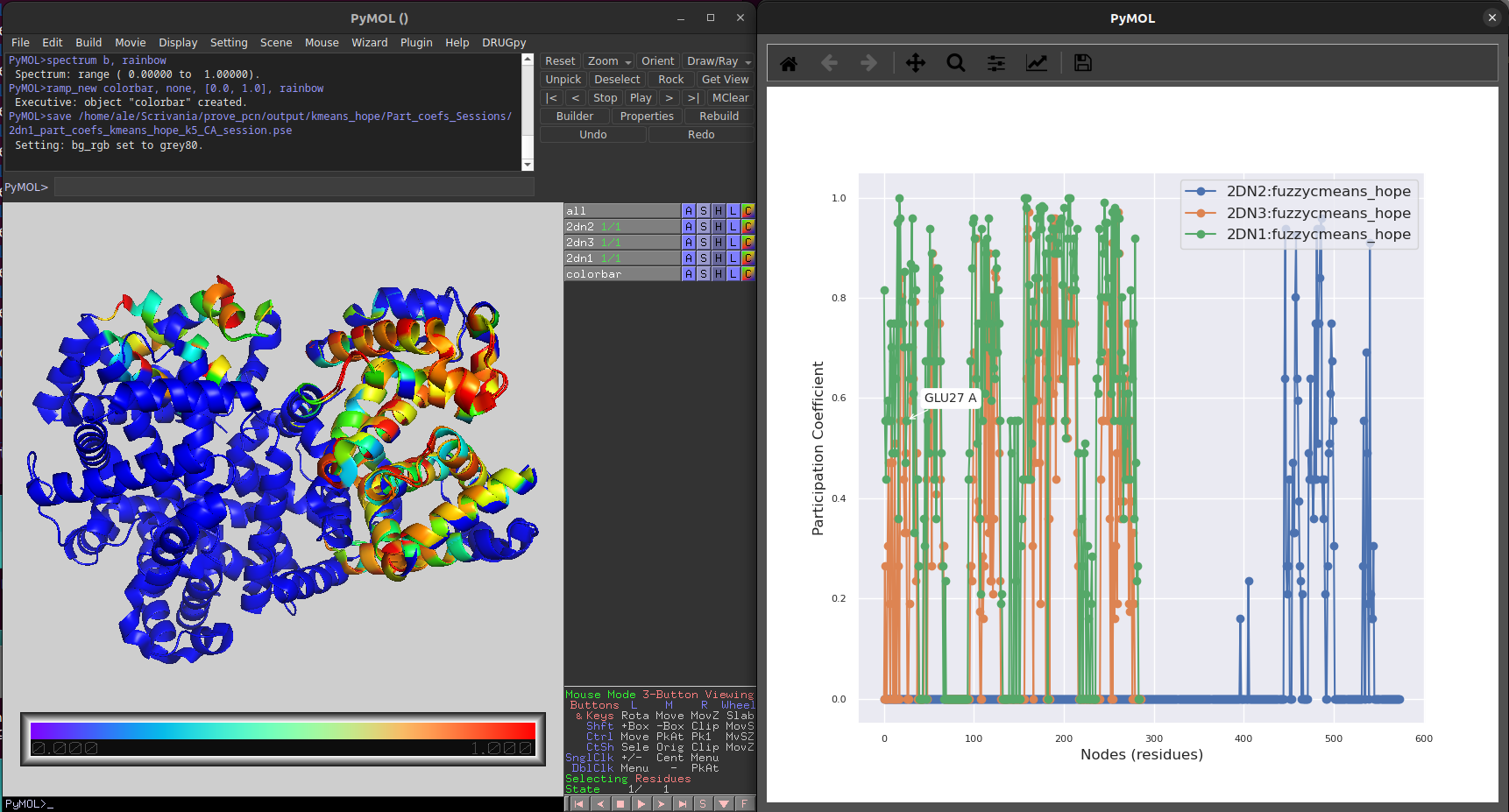


**c**

**Figure 8.2.1** Tutorial - Comparison of hemoglobin oxygen-bound states

## 8.3 - Centroids-based PCN on Aurora-A kinase in complex with its activator TPX-2 (PDB-ID: 1OL5)

This is a use case in which we will explore the usage of PyPCN for analyzing a protein complex, through the computation of a centroid-based adjacency matrix

1. **INPUTS tab (Figure 8.3.1-a)**

The PDB-IDs of interest is 1OL5. The PDB-ID corresponds to the protein to be analyzed, without any modification required. Therefore, we can directly Insert the PDB-IDs (“1ol5”) in the input prompt named as *‘PyMOL object name or PDB-ID’*.

For this analysis, we will proceed with computing the adjacency matrix according to residues’ centroids, by selecting this option in *‘Adjacency Matrix Type’*

1. **COMMUNITY EXTRACTION tab**

- Check one or multiple preferred algorithms. In this example we will use the louvain algorithm, hence the option ‘louvain’ is checked (See Section 9.2).
- Click on ‘Run Analysis’

1. **RESULTS tab**

- To visualize the clustering partition in PyMOL, scroll to ‘1ol5_Communities_louvain_ncoms7_sessioncentroid.pse’ in the section ‘COMMUNITY EXTRACTION’ and click on ‘View in PyMOL’ **(Figure 8.3.1-b,c).**
- To visualize the interactive contact map, scroll to ‘1ol5_adj_centroid_4_8.txt’ and click on ‘Show Contact Map’. By clicking on the map you can explore the significant contacts **(Figure 8.3.1-d)**.


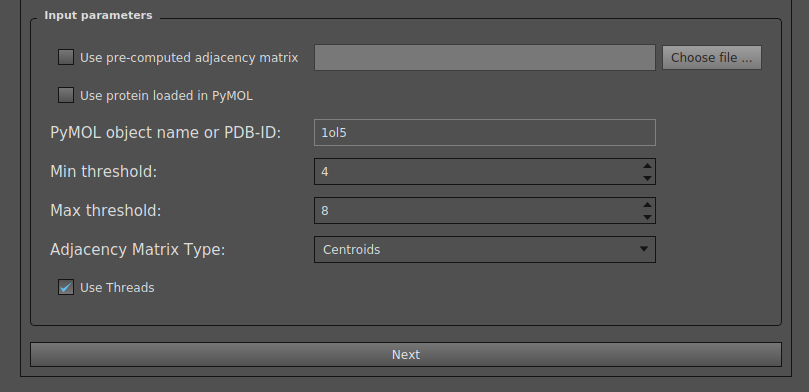


**a**


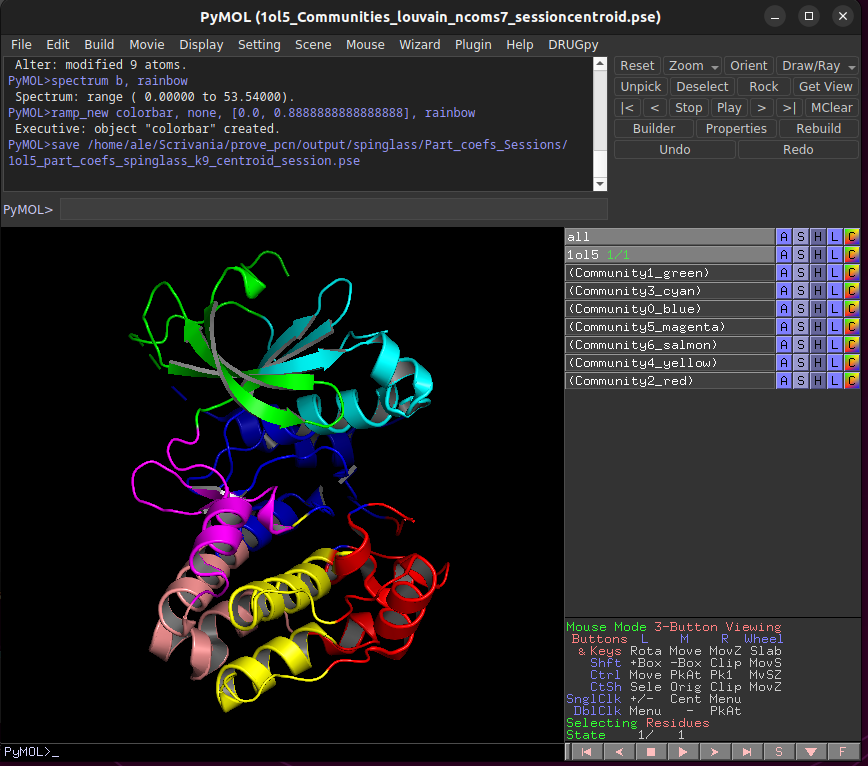

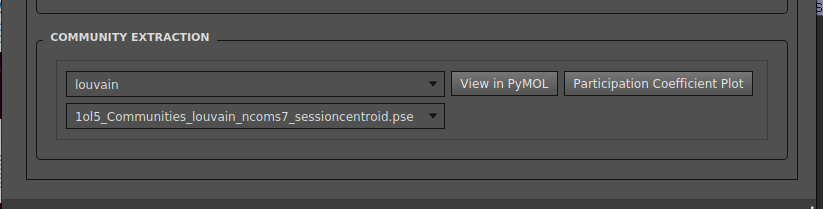


**b**


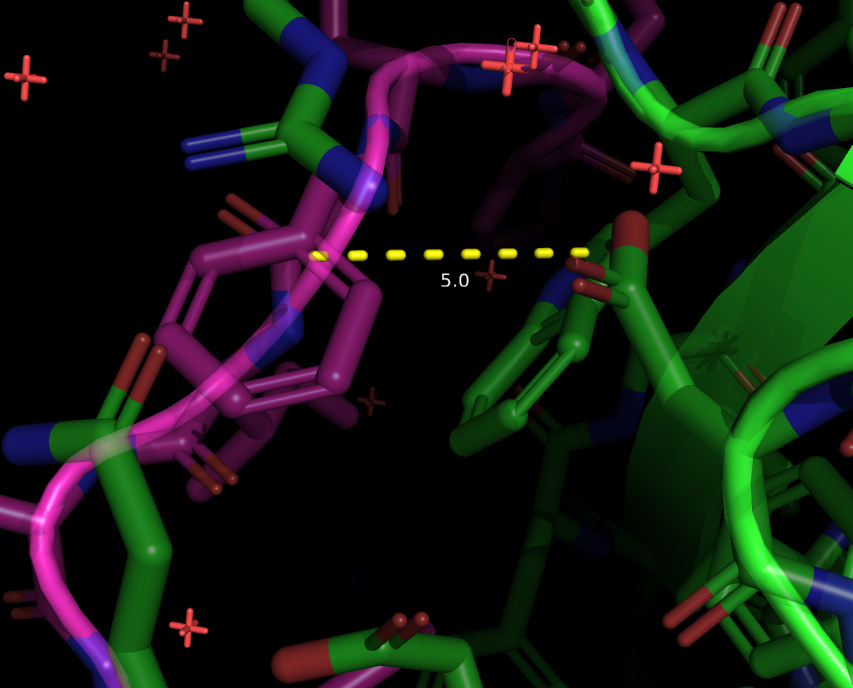


**c**

**d**

**Figure 8.3.1** Tutorial - Centroids-based PCN on Aurora-A kinase in complex with its activator TPX-2 (PDB-ID: 1OL5)

## 8.4 - Conformational ensembles analysis exploiting PCNs

**Note:** The functionalities outlined in this tutorial need pre-computed inputs derived from a simulated conformational ensemble, as detailed in the referenced publication [https://pubs.acs.org/doi/full/10.1021/acs.jcim.8b00250]. To facilitate user adoption and understanding, we have made available sample files extracted from the referenced work. These sample files serve as illustrative inputs to accompany the tutorial and enable users to explore the described functionalities with ease. We encourage users to refer to the specified publication for comprehensive details on the generation and preparation of these simulated conformational ensemble inputs, ensuring a clear understanding of the methodology and context underlying the tutorial.

1. **Download:**

- ‘simulation_1.txt’: <https://github.com/pcnproject/PyPCN/releases/download/utilities/simulation_1.txt>
- ‘simulation_2.txt’: <https://github.com/pcnproject/PyPCN/releases/download/utilities/simulation_2.txt>
- ‘reference_pdb.pdb’: <https://github.com/pcnproject/PyPCN/releases/download/utilities/reference_pdb.pdb>

1. **DCNA tab (‘Other’) - Input files**

- Insert input files in the section ‘Map Contacts’ of the two simulations to compare (‘Simulation 1’ and ‘Simulation 2’) and of the PDB file of interest (‘Open PDB file’)
- [optional] Modify the standard parameters in the ‘Advanced Options’ section.

1. **DCNA tab (‘Other’) - Run analysis**

- Click on ‘Compute Consensus’ and ‘Compute Difference’ to compute, respectively, the consensus contact map and the difference contact map.
- Explore the results by clicking on ‘Show Contact Map’ and ‘Map in PyMOL’ buttons.

1. **INPUTS tab**

- Check the option *‘Use protein loaded in PyMOL’*.
- Insert the PyMOL object name (“reference_pdb”) in the input prompt named as *‘PyMOL object name or PDB-ID’*.
- Disable ‘Use Threads’ if necessary (see Note in Section 6.1)

1. **Centrality, Clustering and communities detection.**

Explore the other tabs to continue further with analyzing the protein of interest (“reference_pdb”) as explained in the other tutorials.


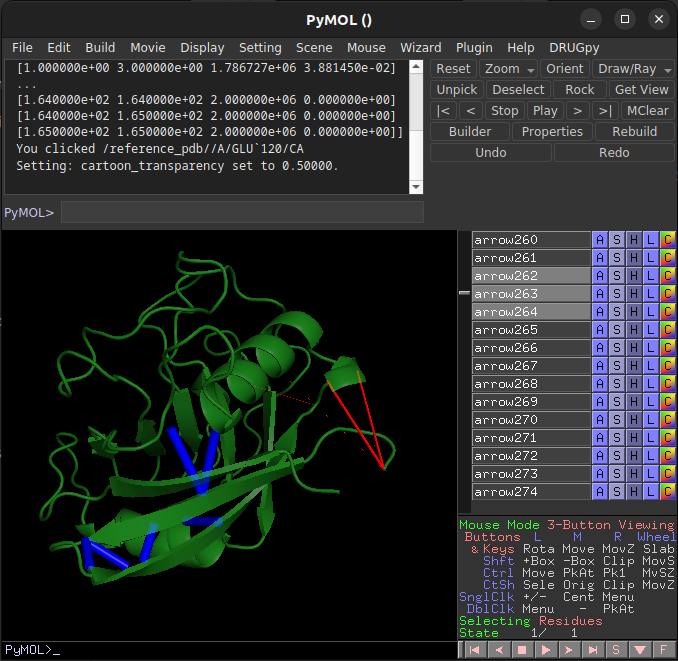

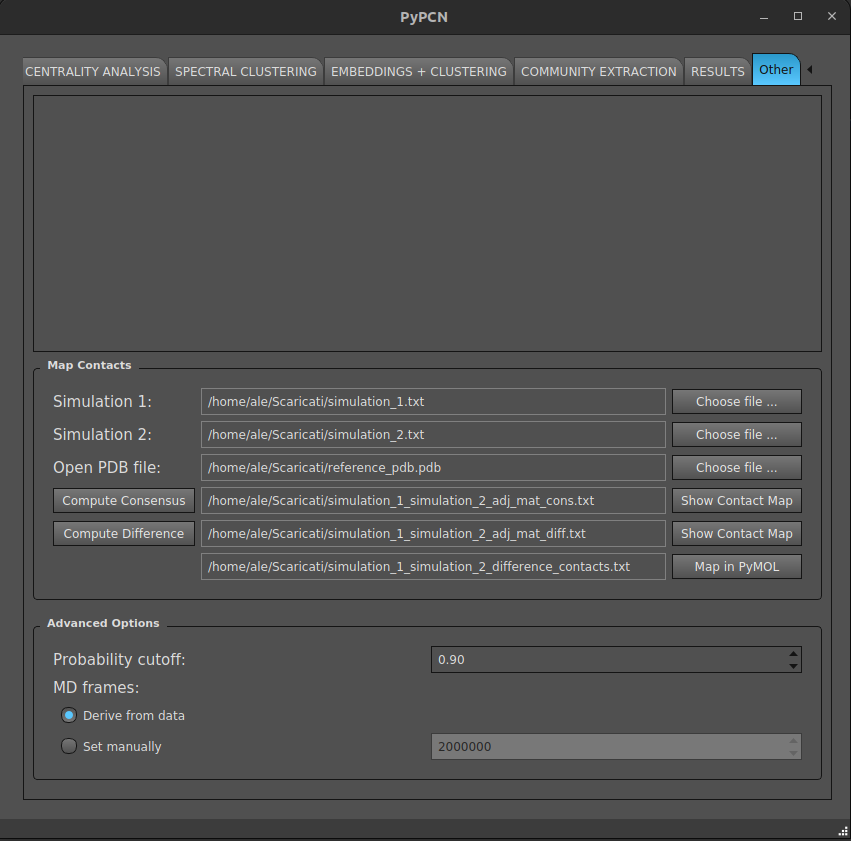


**a**

**b**

**Figure 8.4.1** Tutorial - Conformational ensembles analysis exploiting PCNs

# 9. Supporting material

## 9.1 - External files formats

**9.1.1 - Pre-computed adjacency matrix**

An example of a formatted ‘.txt’ file that stores a pre-computed adjacency matrix can be found at: <https://github.com/pcnproject/PyPCN/releases/download/utilities/precomp_adj.txt>

**9.1.2 - Conformational ensemble statistics**

Examples of input files can be found at:

- ‘simulation_1.txt’: <https://github.com/pcnproject/PyPCN/releases/download/utilities/simulation_1.txt>
- ‘simulation_2.txt’: <https://github.com/pcnproject/PyPCN/releases/download/utilities/simulation_2.txt>
- ‘reference_pdb.pdb’: <https://github.com/pcnproject/PyPCN/releases/download/utilities/reference_pdb.pdb>

PyPCN needs the results of two MD simulations modified to have a three-columns file, in which the first two columns refer to couples of residues and the third column contains the number of frames in which the two residues have been in contact.

## 9.2 - Algorithms and Metrics

In this section is reported a quick summary of the metrics implemented in PyPCN and their meaning in the context of PCNs. This is intended as a general introduction, for further details, it is advisable to consult the original references.

**Node degree**

The Node degree is a measure of the number of links involving a node.

$$k_{i}=\sum_{j} {Ad}_{ij}$$

$${Ad}_{ij}\{1, min\leq d_{ij}\leq max 0, else$$

${Ad}_{ij}$ is 1 if nodes ***i*** and ***j*** are connected by a link; otherwise it is 0;

$d_{ij}$ is the *Euclidean distance*.

The shortest path, between two nodes, is the minimum number of edges connecting them. As the average shortest path lowers, the efficiency of signal transmission increases. In PCNs it correlates with the protein attitude to allosteric regulation (Di Paola et al., 2015; Hu et al., 2017, De Ruvo et al., 2012).

**CENTRALITY MEASURES**

High node centralities in a PCN are associated with residues that are pivotal for protein folding and stability, which likely are also highly conserved (De Ruvo et al., 2012; del Sol et al., 2006).

**Betweenness centrality**

The betweenness centrality of a node describes the number of shortest paths passing by it. On a set of vertices, the betweenness centrality of node is defined as follows:

$$betw\left( i \right)= \sum_{v\in V, v\neq i} \sum_{u\in V,u\neq i} \frac{\sigma_{v,u(i)}}{\sigma_{v,u}}$$

$\sigma_{v,u}$  is the total number of the shortest paths connecting two nodes; $\sigma_{v,u(i)}$represents the number of shortest paths connecting the nodes $v$ and $u$ passing on i as well. Since nodes with high values of betweenness centrality take part in many shortest paths, their modification likely influences the network.

**Closeness centrality**

Closeness centrality is to be considered as a central node close to the others in terms of distance. The closeness centrality of node $w_{i}$ is the reciprocal of the average shortest path to $w_{i}$ over all $n-1$ reachable nodes, i.e.

$$C_{\text{closeness }}\left( w_{i} \right)=\frac{n-1}{\sum_{j=1}^{j=n-1,j\neq i} d\left( w_{i},w_{j} \right)}$$

Where $d\left( w_{i},w_{j} \right)$ is the shortest path between $w_{i}$ and $w_{j}$.

Closeness centrality of residues in PCNs has been demonstrated to correlate with the characterization of how a protein can be subject to perturbation by means of auto-regulation mechanisms or external stimuli; residues in the active site of enzymes show both high degree and closeness centrality.

**Eigenvector centrality**

Eigenvector centrality assesses a node's significance within a network by assigning scores based on the principle that nodes with higher scores have a more substantial impact on the node's score than those with lower scores. Research has demonstrated that eigenvector centrality is effective in identifying the involvement of specific residues in transmitting allosteric signals, both at a local and global level (Negre et al., 2018).

Given an unweighted undirected graph $G$ and its adjacency matrix $A$ we can estimate the Eigenvector centrality ($x_{v}$) for each node $v$as

$$x_{v}=\frac{1}{\lambda}\sum_{w\in\backslash Neigh\left( v \right)} x_{w}=\frac{1}{\lambda}\sum_{w\in\mathcal{G}} \mathcal{A}_{\mathcal{v,w}}x_{w}$$

where $\mathrm{Neigh}\left( v \right)$is the set of neighbors of $v$, and $\lambda$ is a constant. The previous equation may be written as in vector notation as the eigenvector equation $Ax=\lambda x$, where $\lambda$ is an eingenvalue for which a non-zero eigenvector solution exists.

**CLUSTERING**

The impact of clustering detection on PCNs relies on the identification of structural motifs, domains or folds, which are singularly functional for the protein (Brinda et al., 2005). Once the network is clustered, different statistical metrics (participation coefficient and intramodule connectivity ZScore) can be computed to quantify how connected is each node both within its own community and to other communities.

**Participation coefficient**

$$P_{i}=1-\left( \frac{K_{si}}{K_{i}} \right)^{2}$$

$K_{i}$ is the overall degree of the node;

$K_{si}$ is the node degree in its own cluster.

A complementary descriptor is the *intramodule connectivity* $Zscore$, defined as follows:

$$Z_{i}=\frac{K_{si}-\underline{K}_{si}}{{SD}_{si}}$$

$\underline{K}$ is the average value and $SD$ the standard deviation, of the degree $K$ extended to the whole network. The intramodule connectivity $Zscore$ quantifies the preference of nodes to connect with nodes in their own clusters. A node with Participation coefficient $P$  value higher than 0.75 has most of its edges connected with residues of other clusters. Therefore, nodes with high values of $P$ have a higher likelihood that the signaling pathways between clusters pass by them. The $P-Z$ maps show a peculiar shape (“dentist’s chair”) for PCNs.

**Spectral clustering**

Spectral clustering extracts clusters from the Laplacian Matrix (***L***), which is derived as: $L=D-A$; where ***D*** is the degree diagonal matrix and ***A*** is the adjacency matrix.

Spectral clustering is implemented in four of its alternatives, ‘Hard’, ‘Soft’, ‘Normalized’ and ‘Not Normalized’, which are respectively denoted in PyPCN with the tag ‘hsc’, ‘ssc’, ‘norm’ and ‘unnorm’. Additionally, the Shi Malik approach is implemented, and is denoted with ‘shimalik’ tag.

**Embedded clustering**

This approach exploits a combination of embedding algorithms and clustering approaches. The algorithms supported for the embedding are Node2Vec, HOPE and Laplacian-eigenmap (from the GEM library; https://github.com/palash1992/GEM). The combinations with ‘fuzzy-c-means’ and ‘k-means’ clustering, create the 6 solutions implemented in PyPCN.

**Community extraction**

This approach exploits the algorithms of the ‘cdlib’ library (https://cdlib.readthedocs.io/en/latest/), i.e. Louvain, Leiden, Walktrap, Infomap, Asyn FluidC, Greedy Modularity and Spinglass.

**CONFORMATIONAL ENSEMBLE STATISTICS**

From the MD simulations file formatted as explained in section 9.1.2, PyPCN computes:

- The probability of occurring contacts, independently for both simulations;
- The consensus contact map: according to a probability cutoff value, the two simulations are compared for contacts occurring in both.
- The difference contact map, by subtracting contact probabilities of one simulation from their counterparts of the other.

These analyses have been adapted from: <https://pubs.acs.org/doi/full/10.1021/acs.jcim.8b00250>

To facilitate user adoption and understanding, we have made available sample files extracted from the referenced work. These sample files serve as illustrative inputs to accompany the tutorial and enable users to explore the described functionalities with ease. We encourage users to refer to the specified publication for comprehensive details on the generation and preparation of these simulated conformational ensemble inputs, ensuring a clear understanding of the methodology and context underlying the tutorial.

# References

· Brinda KV, Vishveshwara S. A network representation of protein structures: implications for protein stability. Biophys J. 2005;89(6):4159-4170. doi:10.1529/biophysj.105.064485

· Di Paola L, Giuliani A. Protein contact network topology: a natural language for allostery. Curr Opin Struct Biol. 2015;31:43-48. doi:10.1016/j.sbi.2015.03.001

· De Ruvo M, Giuliani A, Paci P, Santoni D, Di Paola L. Shedding light on protein-ligand binding by graph theory: the topological nature of allostery. Biophys Chem. 2012;165-166:21-29. doi:10.1016/j.bpc.2012.03.001

· De Ruvo M, Giuliani A, Paci P, Santoni D, Di Paola L. Shedding light on protein-ligand binding by graph theory: the topological nature of allostery. Biophys Chem. 2012;165-166:21-29. doi:10.1016/j.bpc.2012.03.001

· del Sol A, Fujihashi H, Amoros D, Nussinov R. Residues crucial for maintaining short paths in network communication mediate signaling in proteins. Mol Syst Biol. 2006;2:2006.0019. doi:10.1038/msb4100063

· Hu G, Di Paola L, Liang Z, Giuliani A. Comparative Study of Elastic Network Model and Protein Contact Network for Protein Complexes: The Hemoglobin Case. Biomed Res Int. 2017;2017:2483264. doi:10.1155/2017/2483264

· Negre, C. F. et al. Eigenvector centrality for characterization of protein allosteric pathways. Proc. Natl. Acad. Sci. 115, E12201–E12208 (2018)
